# Evaluation of the effect of fatigue on the coping behavior of international truck drivers

**DOI:** 10.1186/s40359-020-00440-2

**Published:** 2020-07-06

**Authors:** Siamak Pourabdian, Saeid Lotfi, Saeid Yazdanirad, Parastoo Golshiri, Akbar Hassanzadeh

**Affiliations:** 1 Department of Occupational Health Engineering, School of health, Isfahan University of medical sciences, Isfahan, Iran; 2 Department of Occupational Health Engineering, School of health, Tehran University of medical sciences, Tehran, Iran; 3 Department of community medicine and family physician, School of medicine, Isfahan University of medical sciences, Isfahan, Iran; 4 Department of Statistics and Epidemiology, School of Health, Isfahan University of Medical Sciences, Isfahan, Iran

**Keywords:** Fatigue, Coping behavior, International truck drivers

## Abstract

**Background:**

Fatigue can affect the behavior of drivers. While the driver must be able to respond and cope appropriately to the critical situations, which is known as the ability to cope with a crisis. It is likely that the fatigue can change the people’s coping style and thereby increase the chance of the crashes. Therefore, the present study aimed to investigate the effects of fatigue on the coping behavior of international truck drivers.

**Methods:**

This study was conducted on 239 of international truck drivers employed in Iran. The Endler and Parker coping strategies questionnaire (CISS) and Persian version of the Fatigue Multidimensional Fatigue Inventory (MFI) were used to evaluate the coping styles of the drivers and the drivers’ fatigue, respectively.

**Results:**

The mean values of the total fatigue before and after traveling were 36.77 and 76.13, respectively. The mean values of coping styles of the problem-oriented, emotion-oriented, and avoidance before traveling were 53.66, 40.91, and 38.17, respectively, and those after traveling were 45.59, 51.18, and 36.45, respectively. The chi-square test demonstrated that there was a significant difference in the coping style of drivers before and after the trip (*P* <  0.001), and the percent of individuals with emotion-oriented increased.

**Conclusions:**

In general, the results showed that fatigue due to traveling could change the coping styles of subjects from problem-oriented to emotion-oriented and avoidance. This can increase the statistics of driving accidents.

## Background

### Traffic crashes statistics

Traffic crashes are one of the most important health problems in both developing and developed countries. According to a report published in 2002 by the World Health Organization (WHO), every year more than 1.25 million people are killed in road accidents, and more than 50 million people are injured seriously [1]. Driving-related accidents cost about 518 billion dollars globally [2]. Deaths caused due to traffic crashes in Iran are among the highest in the world and have accounted for almost four times the global standard [3]. Based on the reports from Iran’s Forensic Medicine Organization, during 12 months of 2012, 2013, 2014, 2015, 2016, and 2017, respectively, 19,089, 17,994, 16,872, 16,582, 15,932 and 16,201 people died from traffic crashes [3]. The economic costs of driving-related accidents in underdeveloped countries ranged from 1 to 1.5% of the gross national product, and in developed countries, this was 2% [2]. However, the problem is not limited only to the number of people killed because for each death due to traffic crashes, three people are left disabled, and ten people are injured [4].

### Fatigue due to driving

Many factors can affect traffic crashes. One of the most effective agents in occurrence of accidents is human fatigue. Based on the statistics of the police in Iran, the cause of 80 % of the traffic crashes is related to the human agents that include fatigue and sleepiness, deviation to the left, lack of attention, and speed [3]. The exact definition of fatigue is difficult to state due to its complexity and quality. However, fatigue may be described as a condition in which the capacity and willingness to work, and human activity are reduced. In fact, the person’s performance becomes slower and less efficient, and one lacks the capability to do physical or intellectual work [5]. Fatigue is a multidimensional feeling that includes the physical dimension (lack of energy, and need for rest), cognitive dimension (significant decrease in the focus of senses and attention), and emotional dimension (decrease in motivation) [6]. The study by Gataldi et al. (2014) demonstrated that fatigue due to long-duration driving affects the performance of the drivers, and increases the likelihood of accidents and pseudo-accidents [7]. Williams and Boufous (2006) resulted that fatigue-involved traffic crashes are more likely to cause fatality [8]. It seems that driving fatigue is more serious and dangerous in professional drivers, such as truck drivers [9]. The truck drivers are mainly susceptible to fatigue damage because of potential sleep loss, prolonged driving periods and distance, circadian rhythm disruption, and relatively high time pressure journeys [10, 11]. Perttula et al. (2011) concluded that long working shifts, short sleep, and improper break times significantly enhanced the risk of fatigue in heavy vehicle drivers [12]. Also, professional driving is associated with stress-related to psychosocial factors, such as job strain, effort-imbalance, and social support at work. Useche et al. (2017) founded that fatigue links the stress related to working conditions with risky driving in bus rapid transport drivers [13]. The fatigue increases the risk of traffic crashes in professional drivers. The results of a study performed by Lynn and Lockwood (1998) revealed that professional drivers compared to the general public are 49% more likely to be involved in traffic crashes [14]. The results of a study indicated that 18.6% of single truck accidents have occurred because of overfatigue and falling asleep [15]. Fatigue can affect people’s driving in various ways and lead to accidents. The results of Philip et al. (2005) study showed that the sleepiness combined with fatigue significantly affected the reaction time and performance of the drivers [16]. In addition, the study by Bordbor et al. (2008) conducted on 400 male drivers also indicated that there is a significant relationship between fatigue and inappropriate driving behavior [17]. Therefore, fatigue can affect the behavior in addition to alertness in professional drivers and enhance the risk of traffic crashes. Furthermore, the fatigue influence on the stress. The results of a study carried out by Kocalevent et al. (2011) showed that fatigue had a high association with the perceived stress [18]. It seems that there is a two-sided relationship between fatigue and stress.

### Coping behavior

The driver must be able to respond appropriately to the critical situations and take the right decisions in order to avoid high-risk behaviors in stressful conditions and, consequently prevent disastrous accidents—this is known as the ability to cope with a crisis. Individuals are divided into different coping styles based on this feature. These coping styles include the problem-oriented people, who attempt to solve a problem purposefully and logically; the emotion-oriented individuals, who rely on emotional reactions instead of its logical solution and the data processing; and the avoidance individuals, who try to reduce the psychological pressure by avoiding problem [19]. Verma et al. (2017), concluded that emotion-oriented people had higher chances of being involved in accidents [20]. As well as, Lotfi et al. (2017) showed that the individuals having emotion-oriented coping style demonstrated a more dangerous driving behavior, and thus are involved in more car crashes [21].

It is likely that the fatigue can change the people coping style from the problem-oriented to the emotional-oriented and avoidance and thereby increase the chance of the crashes. Therefore, the present study aimed to investigate the effects of fatigue on the coping behavior of international truck drivers.

## Methods

It was assumed in the present study that the fatigue due to commercial trips decrease the coping style of the problem-oriented and increase the styles of emotional-oriented and avoidance in the individuals. The research method was designed to examine this hypothesis.

### Participants

This study was conducted on 239 of international truck drivers employed in Iran in summer of 2017. This study was carried out with the help of the traffic police of Iran, and the unit of violations and road accidents of Iran. For the selection of drivers, researchers inhabited in the traffic police office of the transition border of Iran-Iraq in Ilam for 4 weeks and invited the truck drivers to participate in the study. The sampling was done using the simple random sampling method. The inclusion criterion for the study were males over 30 years old, driving license in grade one, 5 years of job experience, non-addiction license for stimulants such as alcohol, opium, cannabis, and methamphetamine, and presence of any well-known mental illnesses in the participating drivers. The driving behavior is different between males and females. Schwebel et al. (2006) concluded that there were differences between man and woman in risk-taking driving behavior [22]. However, women were excluded due to their very low representation in the occupation of truck driving in Iran.

### Procedure

The volunteer drivers were screened by an expert regarding to inclusion criterion and were requested to confirm the consent from for the participation in this study. The demographic information of participants including age was obtained using the questionnaire. Then, the same drivers completed the coping style and fatigue questionnaires before and after the trip. For this purpose, the drivers answered to questions (one-time) before traveling. When the drivers were back in Iran after the trip, the time of their return was recorded, and questions related to both questionnaires were asked again. These drivers traveled from Mehran of Iran to Sulaimaniyah of Iraq and from Sulaimaniyah of Iraq to Diyarbakir of Turkey. The averaged path length and driving time duration were 1050 km and 30 h, respectively. In addition, the time duration of the trip was 4 days, and the driver had no driving at night.

### Instruments

At first, the mental health was assessed using the multi-dimensional Minnesota Multiphasic Personality Inventory (MMPI) questionnaire. MMPI questionnaire has 71 question and 11 scales of which three are related to test validity (the L scale, which relates to the referee’s attempt rate for presenting positive description from itself, the F scale which deals with the rate of deviation and exceptional answers by the individual, and the K scale that focuses on individual attempts for denial pathology or exaggeration in pathology), and eight other scales relate to the personality indexes including the HS scale or hypochondria, D or depression, HY or hysteria, Pd or social deviation, Pa or paranoia, Pt or mental weakness, Sc or schizophrenia, and Ma or hypomania. The Coping Inventory for Stressful Situations (CISS) was used to evaluate the coping styles of the drivers; the required information regarding the individuals’ coping styles with respect to critical situations was collected using this questionnaire. This questionnaire is a valid instrument developed by Endler and Parker (1990) that is widely used for the identification and comparison of basic coping styles used by various people in different stressful and critical situations [23]. Many studies have reported that CISS has excellent psychometric properties [24]. In total, the questionnaire consisted of 48 questions that distinguish three basic coping styles with 16 questions for each style, including problem-oriented coping style, emotion-oriented coping style, and avoidance coping style. The answers of these questions were graded according to a 5-points Likert scale starting from 1 (never at all) to 5 (too much), and subjects selected value for each question. Then, the scores related to the questions of each style were summed and the style with the highest score was considered as the dominant coping style that is mostly used by one [25]. The reliability coefficients obtained by Endler and Parker (1990) for the three categories, the problem-oriented coping style, emotion-oriented coping style, and avoidance coping style, were 0.9, 0.85, and 0.82, respectively [23]. In Iran, this scale was tested by Shokri et al. in 2006, and the reliability coefficients were obtained as 0.85, 0.76, and 0.73 for the three coping styles, respectively [26]. In addition, the Persian version of the Fatigue Multidimensional Fatigue Inventory (MFI) was used for the evaluation of the drivers’ fatigue. This questionnaire covers five different dimensions of fatigue, which include general fatigue, physical fatigue, reduced activity, reduced motivation, and mental fatigue. The Multidimensional Fatigue Questionnaire consisted of 20 questions that were scored based on the Likert scale from 0 (“yes, it is completely correct”) to 5 (“it is completely incorrect”), and subsequently, the score of each dimension was calculated by summing the scores of its questions. As well as, the score of the total fatigue was calculated by summing the scores of all dimensions. Each dimension was assessed by four questions. The score of each dimension of fatigue lies between 4 and 20, and the total fatigue score obtained from the scores of the fatigue dimensions lies between 20 and 100. A higher score indicates a higher rate of fatigue [27]. This inventory was presented for the first time in the study by Smets. Its validity and reliability were also evaluated among various demographic groups, such as cancer patients, radiotherapy patients, and patients with chronic fatigue syndrome, first-year students of psychology and medicine, soldiers, and students in the third year of medical studies. The alpha coefficient was higher than 0.8 for general, physical, and mental fatigue, and was higher than 0.65 for reduced activity and motivation. The confirmatory factor analysis showed that the questions related to each dimension were their respective descriptors, and the inventory had a proper internal consistency [28]. In Iran, this scale was tested by Hafezie et al. in 2010, and its reliability was estimated at 0.85 [29].

### Data analysis

**Fig. 1:**
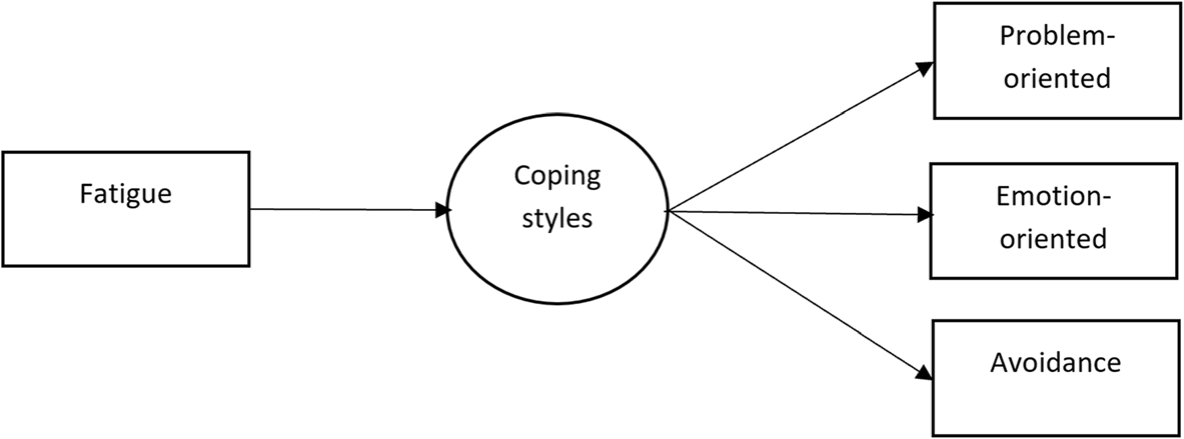
The baseline theatrical model

Finally, the collected information was extracted from the inventories and analyzed using the paired sample t test, chi square test, and ANOVA statistical tests using the SPSS software (version 18). Also, the Pearson correlation coefficient between the total fatigue and each of the coping styles variables was calculated. In addition, two models were drawn using Amos software (version 24) to investigate the effect on the total fatigue on the coping styles based on the Structural equation modeling (SEM) analysis. Figure 1 shows the baseline theatrical model used in the present study. The models were modified based on the modification indices, and standardized estimates were computed. The discrepancy in the models was based on the maximum likelihood. The minimum acceptable significance level was considered by 0.05. The fitness of the final models was also examined using absolute indices (goodness-of-fit index (GFI) and adjusted goodness-of-fit index (AGFI)), comparative indices (normed fit index (NFI), comparative fit index (CFI), and incremental fit index (IFI)), and normed fit indices (root mean squared error of approximation (RMSEA) and normed chi-square (X^2^/df)).

### Ethics statement

The protocol of this study was reviewed and approved by the Medical Ethics Committee of Isfahan University of Medical Sciences. All steps of the study were in accordance with the ethical standards. All participants were asked to fill out the consent form developed by the ethics committee of Isfahan University of Medical Sciences, and written informed consent was obtained from all of them.

## Results

The mean age of the participants was 43.42, and their standard deviation was 8.18. The mean and standard deviation of the fatigue dimensions and the total fatigue scores related to the drivers (before and after an international commercial trip) are shown in Table 1. The statistical test results of the t-test and paired t-test indicated that there was a significant difference in the fatigue score before and after the trip (*p* <  0.001). The mean ​​and standard deviation values of the coping styles of the drivers before and after an international commercial trip are shown in Table 2. The statistical test results of the sample paired t-test indicated that there was a significant difference between the coping styles scores before and after the international trip (*p* <  0.001).

**Table 1:** The mean and standard deviation of the fatigue dimensions and the total fatigue scores related to the drivers before and after an international commercial trip

Parameters	Before an international commercial trip		After an international commercial trip		*p* value
	Mean	Standard deviation	Mean	Standard deviation	
General fatigue	5.34	1.36	17.39	2.14	< 0.001
Physical fatigue	5.93	1.46	16.06	2.31	< 0.001
Reduced activity	9.81	2.12	12.87	2.69	< 0.001
Reduced motivation	6.57	1.91	16.24	2.69	< 0.001
Mental fatigue	5.71	1.43	16.62	2.75	< 0.001
Total fatigue	36.44	5.13	76.13	8.23	< 0.001

**Table 2:** The mean and standard deviation values of the coping styles of the drivers before and after an international commercial trip

Parameters	Before an international commercial trip		After an international commercial trip		*p* value
	Mean	Standard deviation	Mean	Standard deviation	
Problem-oriented	53.66	14.00	45.59	12.50	< 0.001
Emotion-oriented	40.91	10.75	51.18	11.95	< 0.001
Avoidance	36.45	11.57	38.17	14.57	< 0.001

In the CISS inventory, each person obtained the highest score in one of the three coping styles, which indicated the dominant coping style of the individual. Table 3 shows the frequency along with the relative frequency of the drivers with their dominant coping styles before and after the commercial trip. The chi-square test demonstrated that there was a significant difference in the coping style of drivers before and after the trip (*p* <  0.001).

**Table 3:** The frequency along with the relative frequency of the drivers with their dominant coping styles before and after the commercial trip

Parameters	Before an international commercial trip		After an international commercial trip	
	Frequency	Relative frequency (percent)	Frequency	Relative frequency (percent)
Problem-oriented	130	54.4	91	38.1
Emotion-oriented	64	26.8	107	44.8
Avoidance	41	17.2	45	18.8

As well as, Table 4 presents the Pearson correlation matrix of the studied variables before and after an international commercial trip. The results showed that total fatigue had significant negative and positive correlations with the emotion-oriented and problem-oriented styles, respectively, before the trip. The correlation between total fatigue and avoidance style was not significant before the trip. Based on the results, there was a negative correlation between total fatigue and problem-oriented style and the positive correlations between total fatigue with emotion-oriented and avoidance styles after the trip.

**Table 4:** Pearson correlation matrix of the studied variables before and after an international commercial trip

Variables	Before an international commercial trip				After an international commercial trip			
	1	2	3	4	1	2	3	4
Total fatigue	–				–			
Problem-oriented style	- 0.318**	–			- 0.375**	–		
Emotion-oriented style	0.178**	- 0.591**	–		0.285**	- 0.538**	–	
Avoidance style	- 0.034	0.119	- 0.333**	–	0.198**	- 0.295**	0.009	–

Total fatigue, (2) Problem-oriented style, (3) Emotion-oriented style, (4) Avoidance style.

**p < 0.01

**Fig. 2:**
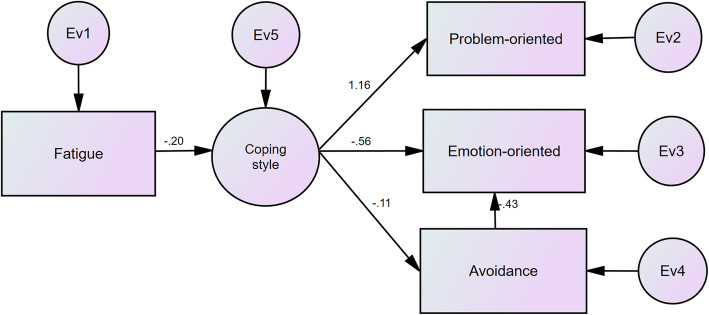
The results of SEM analyses before an international commercial trip

**Fig. 3:**
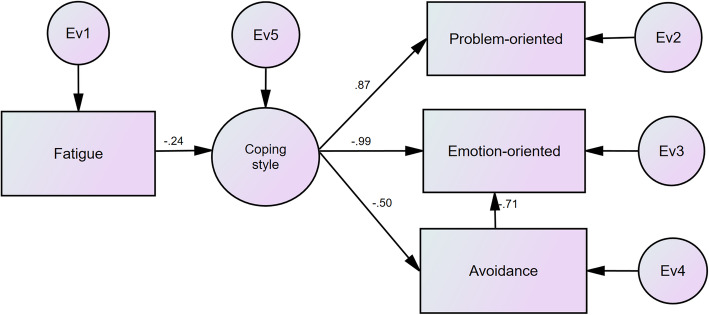
The results of SEM analyses after an international commercial trip

Figures 2 and 3 depict the results of SEM analyses before and after the trip, respectively. Based on the results, the impact coefficient of the total fatigue on the coping style has increased after the trip compared to that before the trip. Also, the factor loading of the problem-oriented style has decreased after the trip than that before the trip, while the factor loadings of the emotion-oriented and the avoidance styles increased after the trip compared to that before the trip. Moreover, the avoidance style had a negative effect on emotion-oriented. This effect enhanced after the trip. Tables 5 and 6 report the goodness-of-fitness indices of the studied models before and after the trip, respectively. The results showed that the calculated values of goodness-of-fitness indices in both models were greater than the values of threshold of fitness. Therefore, the fitness of the models was confirmed.

**Table 5:** The goodness-of-fitness indices of the studied model before the commercial trip

Index	Name	Threshold of fitness	Obtained value
Absolute fitness indices	Goodness-of-fit index (GFI)	> 0.9	0.997
	Adjusted goodness-of-fit index (AGFI)	> 0.9	0.980
Comparative fitness indices	Normed fit index (NFI)	> 0.9	0.992
	Comparative fit index (CFI)	> 0.9	0.998
	Incremental fit index (IFI)	0–1	0.998
Normed fit index	Root mean squared error of approximation (RMSEA)	< 0.1	0.009
	Normed chi-square (X2/df)	1–3	1.102
*p* value		> 0.05	0.941

**Table 6:** The goodness-of-fitness indices of the studied model after the commercial trip

Index	Name	Threshold of fitness	Obtained value
Absolute fitness indices	Goodness-of-fit index (GFI)	> 0.9	0.994
	Adjusted goodness-of-fit index (AGFI)	> 0.9	0.971
Comparative fitness indices	Normed fit index (NFI)	> 0.9	0.989
	Comparative fit index (CFI)	> 0.9	0.995
	Incremental fit index (IFI)	0–1	0.994
Normed fit index	Root mean squared error of approximation (RMSEA)	< 0.1	0.013
	Normed chi-square (X2/df)	1–3	1.365
*p* value		> 0.05	0.546

The mean and standard deviation of the participants’ ages based on their dominant coping style has been shown in Table 7. The results of statistical analysis using the one-way ANOVA test indicated that the mean age of the drivers who exhibited a dominant problem-oriented coping style was significantly more in both periods of travel (before and after the commercial trip) (*P* < 0.001) than the drivers who exhibited dominant emotion-oriented and avoidance coping styles. In addition, the mean age of the people with an emotion-oriented coping style did not vary significantly in comparison with the people who exhibited an avoidance coping style with respect to both periods of the trip (before and after traveling) (*p* < 0.169).

**Table 7:** Mean and standard deviation of the ages of the participants based on their dominant coping style before and after the commercial trip

Parameters	Before an international commercial trip			After an international commercial trip		
	N	Mean	Standard deviation	N	Mean	Standard deviation
Age of problem-oriented subjects	130	46.80	7.36	91	49.24	6.60
Age of emotion-oriented subjects	64	39.20	7.83	107	39.34	7.16
Age of avoidance subjects	45	39.73	6.44	40	41.07	6.01

## Discussion

The results of this study showed that the score of the different dimensions of fatigue after the end of the commercial trip was significantly more than at the beginning of the trip. Zhang et al. (2016) concluded that truck drivers with an odds ratio of 1.57 were at high risk of exhibiting fatigue driving behavior [30]. Chang and Chien (2013) also observed that there is a high risk of fatigue in truck drivers [31]. The finding of the present study is consistent with the results of other studies. Based on these results, most values of increase and decrease were related to the dimensions of general fatigue and reduced activity, respectively. Although fatigue enhanced over time, the individuals tried to maintain their activities. For this reason, they stated a lower score of reduced activity compared to other dimensions of fatigue. However, fatigue is a transitional state between awakening and sleeping that results in a lack of consciousness, and thus causes a reduction in the mental and/or physical performance [32]. These decreased performances can significantly enhance traffic crashes. So that fatigue is one of the main reasons that contribute to road accidents; it is the main reason for the high prevalence of driving accidents with respect to trucks and buses [33]. The results of several studies indicate that approximately 20–50% of all road accidents occurred due to fatigue [34]. Various agents create fatigue in professional drivers. Some of them included sleep deprivation, inappropriate rest breaks, prolonged driving times, loneliness, high time pressure journeys, and psychosocial factors [35]. The effect of prolonged driving time on fatigue was investigated in the present study. The results of this study showed that long-duration driving due to international travel can lead to a significant increase in the physical and mental fatigue in individuals. The results of other studies also indicate that this agent significantly affects the fatigue and consequently, performance in the drivers through different mechanisms. The fatigue can reduce the reaction time, consciousness, and concentration, and disrupts all attention-based activities [36]. The result of a study performed by Szeseen kee et al. (2010) based on the physiological change in EEGs waves along with long-term driving showed that with increasing driving time and fatigue, the drivers’ consciousness was reduced, and their performance was disrupted [37]. In 2014, Wang and Pei evaluated the impact of long duration driving on mental performance and fatigue on 33 professional drivers. The results of their study showed that after 2 hours of continuous driving, there was an increase in fatigue, and the mental performance of the drivers was reduced, which affected their attention, reaction, and perception [38]. In addition to performance, fatigue can influence on individual behavior. In fact, fatigue leads to a shortfall in the cognitive ability, a weakness in one’s ability to judge and decide correctly [36]. These impacts can affect the driving behavior of professional drivers. Liu and Wu (2009) concluded that fatigue deteriorated driving behavior [39]. However, while driving, one must be able to take the right decisions through logical solutions to prevent the occurrence of high-risk behaviors and, consequently, adverse accidents. This is called as being capable of coping with a crisis [40]. Based on the results of the present study, the coping style of emotion-oriented, and avoidance in ones increased and the coping style of the problem-oriented decreased. Consequently, the number of individuals who displayed the dominant emotion-oriented and avoidance coping styles after commercial travel was more than those before traveling. However, after the trip, the number of individuals with a dominant problem-oriented coping style had decreased. Also, the results indicated that there was a significant negative correlation between total fatigue with emotion-oriented, and there were significant positive correlations between total fatigue with problem-oriented and avoidance styles after the trip. The results of SEM analyses revealed that the effect of the total fatigue on the coping style was enhanced after the trip. Based on the results, factor loading of the problem-oriented style decreased, and factor loadings of the emotion-oriented and avoidance styles increased after the trip. Therefore, it can be concluded that increasing the amount of fatigue resulted from the trip can significantly decrease the problem-oriented coping style and increase the avoidance and emotion-oriented coping style in the individuals. These results are logical. Because in the problem-oriented coping style, the individual focuses on the problem, collects and processes information in the mind and, after evaluating them, attempts to solve it logically [41], whereas when individuals are not thinking logically, s/he uses an emotional or avoidance approach to solve problems [41]. It seems that people with increasing fatigue employ the emotional-oriented and avoidance coping styles instead of the problem-oriented coping style for solving the crisis due to a reduction in the cognitive performance, and the resulting weakness in the ability to make a decision. Indeed, people diminish the mental load through emotion and avoidance behaviors after increased fatigue. The results of a study performed by Saxby et al. (2013) indicated that the fatigue decreased alertness evaluated by the speed of braking and steering responses to an emergency event [42]. Desmond and Matthews (2009) also concluded that fatigue and stress can affect driving reactions through the use of emotion-focused coping [43]. the results of the present study also indicated that the avoidance style had a negative effect on the emotion-oriented before and after the trip. It may be because of inconsistent attributes of these two types of coping behavior. However, the results of other studies indicate that drivers exhibiting a dominant emotion-oriented and avoidance coping styles have more experiences of road accidents, which can be dangerous. For example, the study conducted by Lotfi et al., in 2017, on 610 drivers regarding the relationship between coping styles and driving behavior showed that individuals with an emotion-oriented and avoidance styles have experienced more car crashes [21]. Moreover, a study conducted by Tao et al., in 2017 in China, on 511 drivers demonstrated that emotion-oriented drivers and risk-takers had a more dangerous driving behavior [44]. In addition to the aforementioned studies, the results of this study also showed that the age of people with problem-oriented coping style was significantly higher than those with avoidance and emotion-oriented coping styles. The study by Durkin et al. on drivers indicated that young drivers tend to behave in a risky manner, since they do not consider speeding as an abnormal behavior [45]. Even official statistics show that the risk of accidents for young people is the highest and twice as high as that of the elderly [45]. Other probably effective factors on the coping styles include gender, education level, smoking, and rest break planning, which were not investigated because of limitations in the present study. It is proposed that the impacts of these factors are evaluated in the next studies.

## Conclusion

In general, the results of this study suggested that with increased fatigue after the end of the international commercial trip, the dominant coping style in some of them changed from the problem-oriented to emotion-oriented. Considering that emotion-oriented people behave more dangerously and have more crashes, it is proposed that long-duration driving be avoided in case of commercial trips that involve longer travels, and measures are to be taken to reduce the fatigue in these drivers, such as cross-road resort, longer stops compared to normal drivers along the road, and so on.

## Data Availability

All data generated and analyzed during the study are included in this article.

## Abbreviations

WHO: World health organization
MMPI: Minnesota multiphasic personality inventory
CISS: The coping inventory for stressful situations
MFI: Fatigue multidimensional fatigue inventory
SEM: Structural equation modeling
